# Optimal Management of the Public and Patients by Pharmacists in the Era of COVID-19: An Evidence-Based Review and Practical Recommendations

**DOI:** 10.3389/fpubh.2021.758325

**Published:** 2022-01-11

**Authors:** Zaiwei Song, Yang Hu, Zhenyu Ren, Guanru Wang, Shuang Liu, Siqian Zheng, Li Yang, Rongsheng Zhao

**Affiliations:** ^1^Department of Pharmacy, Peking University Third Hospital, Beijing, China; ^2^Institute for Drug Evaluation, Peking University Health Science Center, Beijing, China; ^3^Therapeutic Drug Monitoring and Clinical Toxicology Center, Peking University, Beijing, China; ^4^Department of Pharmacy Administration and Clinical Pharmacy, School of Pharmaceutical Sciences, Peking University, Beijing, China

**Keywords:** patient management, pharmaceutical care, pharmacist, coronavirus disease 2019, COVID-19

## Abstract

**Purpose:** Currently, managing the public and patients during the COVID-19 pandemic is constituting a health care challenge worldwide. Patient-oriented management is of crucial importance to promote emergency preparedness and response. This study aims to formulate an integrated pharmacist management strategy of the public and patients and to provide evidence-based and practical references.

**Methods:** Evidence-based review and practical analysis were utilized. First, PubMed, EMBASE and Chinese database were searched. Studies about patient management in major public health emergencies were included. Second, the Chinese experience of patient management was analyzed and identified. Finally, combining evidence-based and practical analysis, the pharmacist management strategy of the public and patients was researched and summarized.

**Results:** Regarding the home quarantine period, pharmacist management services should include medication guidance, guidance on risk monitoring, sanitation measures education, health management guidance and psychological support. Regarding the outpatient visit period, pharmacists should participate in the control of in-hospital infections and provide physician-pharmacist joint clinic services, pharmacy clinic services, medication therapy management, medication consultation services, drug supply guarantee and drug dispensing services. Regarding the hospitalization period, pharmacist management services should include monitoring and evaluating the safety and efficacy of medications, providing strengthened care for special populations and other pharmaceutical care. For non-hospitalized or discharged patients, pharmacist management services should include formulating medication materials and establishing pharmacy management files for discharged patients.

**Conclusion:** An evidence-based, patient-centered and entire-process-integrated pharmacist management strategy of the public and patients is established, which remedies the gaps in the existing patient management and can be implemented to support pharmacists' contributions to COVID-19 pandemic control.

Coronavirus disease 2019 (COVID-19) is characterized by diverse and uncertain transmission modes, high contagiousness, high transmission speed, wide prevalence, and difficulty in prevention, diagnosis and treatment. Up to December 9th, there were more than 260 million confirmed cases around the globe, including more than 5 million deaths (1). At present, the COVID-19 pandemic is still in severe situation, especially with the Omicron variants fuelling the surge around the world, posing a major challenge to the global medical health system. Meanwhile, it has also caused a huge impact on the global society and economy (2). Public and patient management and infection control are still great matters that must be faced and concerned about. An effective and reliable mode of public and patient management is essential to help the world remain and become better prepared, as well as restoring living order back to normal.

During the COVID-19 pandemic, China immediately formulated and implemented a series of effective plans of prevention and control (3–5), and the trend of the COVID-19 outbreak in China has been almost brought under control. Based on the characteristics of COVID-19, the public and patient management process was divided into four parts in China: home quarantine period, outpatient visit period, hospitalization period, and non-hospitalized or discharged patients. During home quarantine, the infection risk of the public should be evaluated and classified based on the epidemiological history and respiratory symptoms, and corresponding measures, such as continuing home quarantine or timely hospital visits, should be taken. At the time of the visit, patients go to routine clinics or fever clinics after precheck and triage. For patients suspected of having COVID-19 infection, a series of in-hospital disposal procedures will be adopted, including routine laboratory testing, imaging inspection, expert consultation, and viral nucleic acid testing. After the outpatient visit, confirmed cases will be admitted to COVID-19-designated hospitals for further hospitalized treatment, and other patients will receive regular treatment or go home for isolation. The detailed mode of management and monitoring of the public and patients is shown in Figure 1.

**Figure 1:**
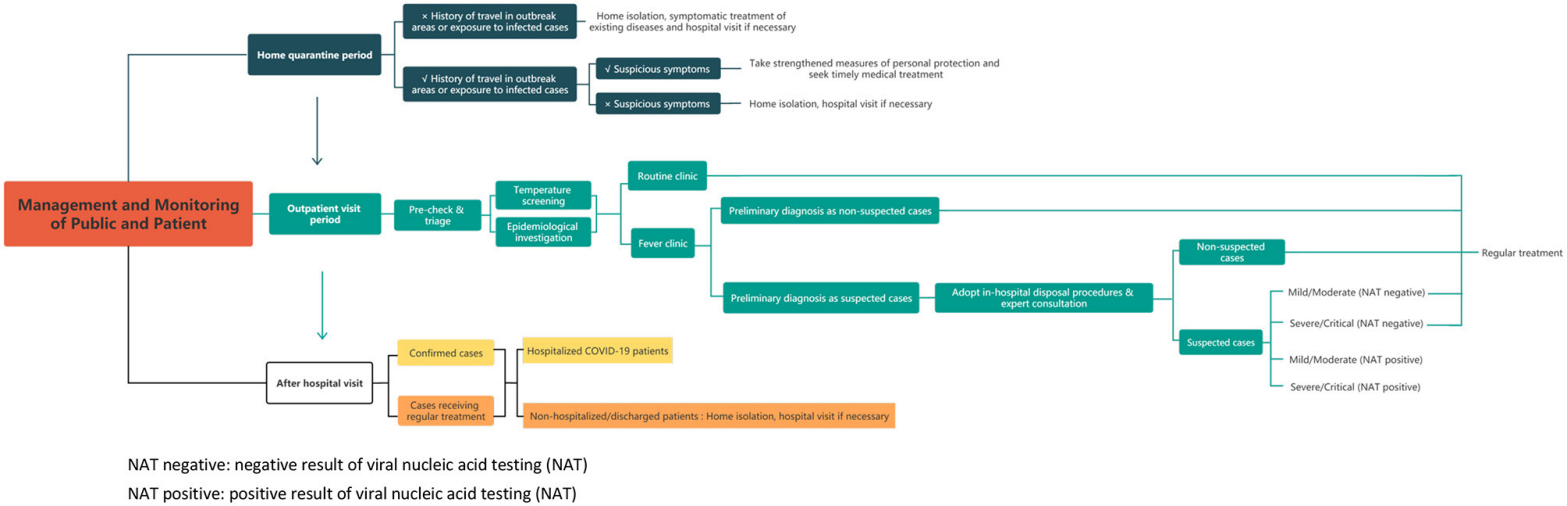
The detailed mode of management and monitoring of public and patient. NAT negative, negative result of viral nucleic acid testing (NAT); NAT positive, positive result of viral nucleic acid testing (NAT).

Medication therapy is one of the most essential forms of treatment. As a member of healthcare professionals, cooperating with medical teams to provide patient management is one of the most important responsibilities for pharmacists (6). In the situation of COVID-19, patients are in high demand of pharmaceutical care in every link of the entire process. In routine medical practice, there has already been a cooperation mode of patient management for clinicians, nurses and pharmacists. However, considerable changes have taken place in patients' medical care during the COVID-19 pandemic, especially in chronic disease management. Patients with chronic diseases are in need of continuous medication. To lower the risk of infection during hospital visits, they tend to reduce the frequency of visits and then seek more medication instruction from pharmacists. Therefore, it is necessary to establish a pharmacist management mode of patients during the COVID-19 pandemic, which covers the entire process of home quarantine and in-hospital treatment.

This study aims to summarize the evidence-based review of patient management research on major public health emergencies and Chinese practical experience of innovative pharmaceutical care during the COVID-19 pandemic and to finally formulate an entire-process integrated strategy for pharmacist management of the public and patients. We hope to provide evidence-based and practical advice for pharmacists to conduct optimal patient management in the COVID-19 pandemic, as well as other public health emergencies in the future.

## Methods

### Evidence-Based Review of Available Evidence

#### Search Strategy

Electronic databases, including PubMed, Embase and Chinese National Knowledge Infrastructure (CNKI), were searched to identify patient management research in major public health emergencies. Specific search strategies were developed for each database. Keywords including *severe acute respiratory syndrome* (*SARS), Middle East respiratory syndrome (MERS), H1N1 influenza, Ebola, Zika, COVID-19, patient management, health care management, patient administration, and patient care* were used to search the title and abstract of the literature.

#### Eligibility Criteria

##### Types of Participants

Suspected or confirmed cases of SARS or MERS or H1N1 influenza or Ebola or Zika or COVID-19 and their close contacts, as well as other patients.

##### Types of Interventions/Comparators

Patient management conducted by clinicians, nurses, pharmacists and other healthcare practitioners. No comparator was set in this study.

##### Types of Outcomes

Patient management strategies designed in the articles.

##### Types of Studies

Studies were considered eligible if their themes were around major public health emergencies, including SARS, MERS, H1N1 influenza, Ebola, Zika and COVID-19. The exclusion criteria were as follows: (1) emergency and protection strategies for medical staff; (2) clinical research; (3) articles written in neither Chinese nor English; (4) other studies with no relation to patient management.

#### Study Identification and Selection

Two investigators (ZS and YH) independently assessed the eligibility of all studies based on the eligibility criteria above after reviewing the study title, abstract and full text in succession. Studies that did not meet the criteria were excluded. Any disagreement among authors was discussed and reconciled by the corresponding author (RZ).

#### Data Extraction and Analysis

Two investigators (ZS and YH) independently extracted data based on a predesigned standardized data extraction form, including the first author, publication year, public health emergency, management providers, managed objects, scope of management, and management measures. A narrative analysis was performed, and an evidence map was used to summarize the patient management strategies of the included studies.

### Formulation of Pharmacist Management Strategy of Public and Patient

Corresponding to the current Chinese mode of management and monitoring of the public and patients in the era of COVID-19 (Figure 1), the demand for pharmaceutical care at every link was analyzed and identified, and innovative pharmaceutical services were fully explored. Finally, the pharmacist management strategy of the public and patients was researched and formulated on the basis of evidence-based review of patient management research on major public health emergencies and practical analysis of Chinese experience during the COVID-19 pandemic.

## Results

### Evidence-Based Review of Patient Management Research on Major Public Health Emergencies

#### Study Selection and Characteristics of the Included Literature

**Figure 2:**
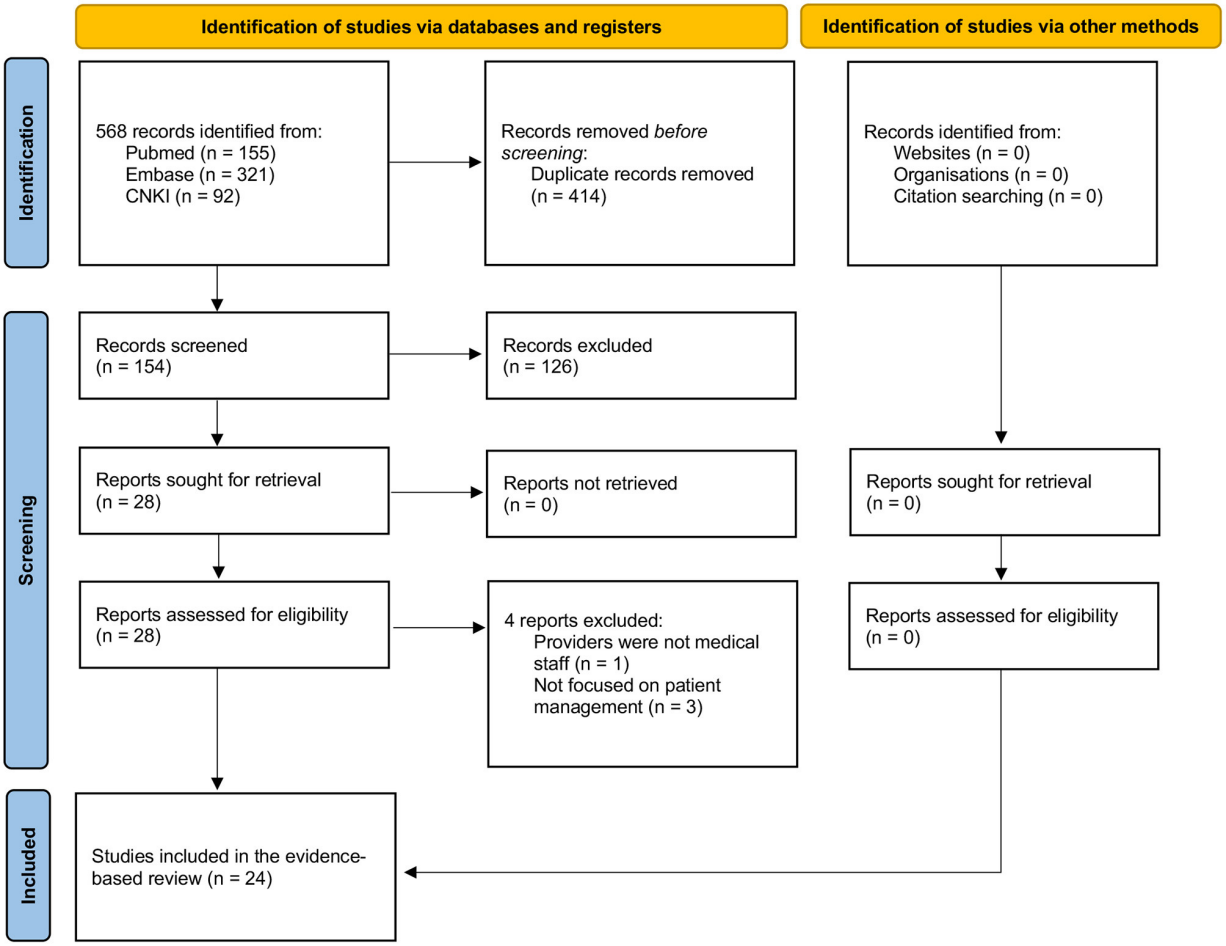
Flow diagram of study selection for the evidence-based review.

A total of 568 candidate references were identified by electronic database searches. Of the total, 414 duplicated references were removed. After careful screening of the references according to the aforementioned eligibility criteria, 24 studies (7–30) were finally included (Figure 2). After data extraction and evidence-based analysis, there were two studies about SARS, one about H1N1 influenza, one about Ebola, one about Zika, and 19 about COVID-19. Of the 19 studies (12–30) on COVID-19, eight studies (12–19) focused on general management, while 11 studies (20–30) focused on the management of patients of clinical specialty under the pandemic situation.

#### Summary of Patient Management Research in Major Public Health Emergencies

**Figure 3:**
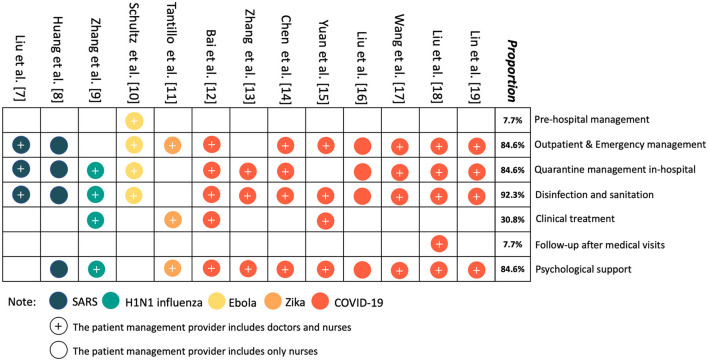
The evidence map of patient management research on major public health emergencies.

The evidence map of studies about patient management in major public health emergencies is shown in Figure 3. Regarding management providers, current studies mainly focus on physicians and nurses, and only one study explicitly mentions the role of pharmacists. Regarding management measures, the included studies mainly focused on modules as follows: prehospital treatment, outpatient and emergency patient management, patient management during isolation, disinfection and cleaning, clinical treatment, patient follow-up, health education and psychological support. There are detailed and feasible measures in most links of the management process, such as strengthening precheck and triage as well as epidemiological investigations for outpatient and emergency patients, isolating suspected patients in a timely manner, and forming an expert group for consultation. For confirmed infected patients, they should be transferred to the isolation ward in a timely manner for further treatment. At the same time, medical staff should strengthen the cleaning and disinfection of medical supplies to reduce the risk of in-hospital infection. In addition, health practitioners should actively provide health guidance and psychological support to patients. However, management measures in some key links of patient management are still relatively weak and urgently need to be strengthened, including self-medication guidance at home (prehospital), monitoring of medication efficacy and safety (in-hospital), and follow-up of discharge (post-hospital).

#### Summary of Patient Management of Clinical Specialties in the COVID-19 Period

Designed to help specialist doctors and nurses provide better patient management in the COVID-19 period, 11 studies (20–30) have been researched and published, involving patients with malignancy, stoma, peritoneal dialysis, chronic viral hepatitis, elderly diabetes, hypertension and pregnant women. Most studies recommend timely diagnosis and referral and quarantine, telemedicine services including online monitoring and regimen adjustment and follow-up, and other targeted management measures. One study explicitly mentions the role of pharmacists, while there are some management measures related to pharmacist involvement in some of the included studies, including establishing medication files, providing medication guidance, monitoring adverse reactions of chemotherapy, implementing long-term prescriptions, and providing drug delivery and other services. The evidence table of studies about patient management of clinical specialties in the era of COVID-19 is summarized in Table 1.

**Table 1:** Evidence table of studies about patient management of clinical specialties during the COVID-19 pandemic.

**References**	**Country**	**Management providers**	**Managed objects**	**Management measures**
Mistretta et al. (20)	Italy	Doctors, Nurses	Patients with malignancy	1. Isolating confirmed patients for further treatment 2. Monitoring the condition after tumor surgery and chemotherapy 3. Online monitoring and prescription adjustment
Xu et al. (21)	China	Doctors, Nurses	Patients with lung cancer	1. Differential diagnosis 2. Monitoring adverse reactions of radiotherapy and chemotherapy 3. Online follow-up and remote monitoring
Ai et al. (22)	China	Doctors, Nurses	Patients with prostate cancer	1. Online medical consultation and long-term prescription 2. Postponement of non-urgent surgery 3. Isolating suspected/confirmed patients for further surgery
Qiu et al. (23)	China	Doctors, Nurses	Patients undergoing ostomy	1. Monitoring the use of ostomy bags, and increase the storage 2. Strengthen nursing and prevention of complications 3. Online monitoring and regimen adjustment
Lai et al. (24)	China	Doctors, Nurses	Patients undergoing peritoneal dialysis	1. Online monitoring and prescription adjustment 2. Isolating suspected/confirmed patients for further treatment
Yang et al. (25)	China	Doctors, Nurses	Patients undergoing peritoneal dialysis	1. Online monitoring and prescription adjustment 2. Isolating inpatients in separate ward
Ronco et al. (26)	Italy	Doctors, Nurses	Patients undergoing peritoneal dialysis	1. Assisting the referral 2. Online monitoring and prescription adjustment 3. Online medical care, and drug delivery 4. Postponement of non-urgent surgery 5. Home-based dialysis guidance
Li et al. (27)	China	Doctors, Nurses, Pharmacists	Patients with chronic viral hepatitis	1. Long-term prescription 2. Online monitoring and regimen adjustment 3. Establishing medication files, and providing medication guidance
Chen et al. (28)	China	Doctors, Nurses	Pregnant and parturient women	1. Taking regular antenatal examination 2. Evaluating the condition of suspected/confirmed patients, and regimen adjustment 3. Suspending lactation for suspected/confirmed patients
Gong et al. (29)	China	Doctors, Nurses	Elderly patients with diabetes	1. Blood glucose monitoring 2. Online follow-up, informationized management of blood glucose 3. Online monitoring and prescription adjustment 4. Strengthen consultation, and assisting the referral
Xiong et al. (30)	China	Doctors, Nurses	Patients with hypertension	1. Online medical care, and drug delivery 2. Tele-medication guidance, and improving compliance 3. Follow-up in a targeted manner

#### Findings About Pharmacist Management Services From Evidence-Based Review

As one of healthcare professionals, there is still a lack of practical guidance in entire-process patient management by pharmacists during major public health emergencies. Prehospital treatment, medical treatment after admission, and patient follow-up are particularly critical to improve the curing rate at an early stage and promote pandemic control. As one of the most accessible healthcare professionals before being admitted, pharmacists should give full play to their expertise to conduct monitoring and follow-up of patients, establish management documents, and assist in referrals when necessary. During admission, pharmacists should cooperate with other healthcare providers to fully participate in clinical treatment, formulate medication regimens based on the patient's condition, and strengthen pharmaceutical care. In addition, since some discharged patients retested positive for nucleic acids, pharmacists should take designed measures for discharged patients to strengthen follow-up.

### Pharmacist Management of the Public and Patients During the COVID-19 Pandemic

**Figure 4:**
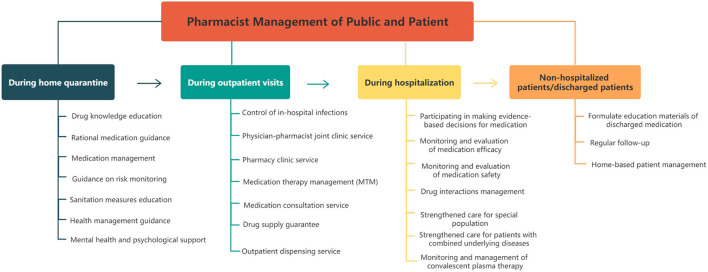
Strategy of pharmacist management of the public and patients during the COVID-19 pandemic.

In the situation of COVID-19, pharmacists should give full play to the role of public health management while providing pharmaceutical care combined with the pandemic. A patient-centered and integrated patient management strategy was researched and established, which covers the entire process, including home quarantine, outpatient visits and hospitalization. See Figure 4 for further details. Notably, regular pharmaceutical care (drug supply guarantee, rational medication guidance, MTM, etc.) as well as public health services (risk monitoring guidance, sanitation measures education, health management guidance, etc.) are attached equal importance in this strategy.

#### Home Quarantine Period

COVID-19 is a highly infectious disease, and Chinese experience has proven that home quarantine is one of the most direct and effective approaches to prevent and control the epidemic. With the rapid pandemic, many countries and regions around the world have initiated the provision of “closing the city” and isolating the public at home. Consequently, the importance of patient management by pharmacists during home isolation is more prominent.

Currently, since most potential drugs for COVID-19 are still in clinical trials, pharmacists should ensure patients not to use drugs on their own following the treatment protocols from unidentified network sources. Pharmacists should provide home-based pharmaceutical care as follows (6): (1) Drug knowledge education. Pharmacists should provide helpful information about the identification of drugs and supplements, identification of prescription and non-prescription drugs, drug preservation, and drug instruction reading. (2) Rational medication guidance. Pharmacists should provide guidance on usage and dosage, dose adjustment for specific populations, and drug interactions (such as cephalosporin and alcohol, lipid-lowering drugs and grapefruit). (3) Medication management. To improve patients' medication compliance, pharmacists should assist patients, especially those with long-term medications, to establish a self-medication therapy record, which shall include basic information, long-term medication plans, and health supplements, temporary medications, monitoring of health indicators, monitoring of discomfort or adverse reactions, etc.

In addition to regular pharmaceutical services, pharmacists should also actively play their roles in public health management during the pandemic as follows: (1) Guidance on risk monitoring (6). Pharmacists should ensure people at home quarantine to conduct active and continuous self-monitoring of suspicious symptoms of COVID-19 (including fever, cough, sore throat, chest tightness, etc.). Additionally, asymptomatic infected individuals should be fully vigilant. (2) Sanitation measures education (31). In addition to providing guidance on cleaning and disinfection of the environment and personal protection, for patients with suspicious symptoms who need to seek medical treatment, pharmacists should apply strengthened management strategies to ensure that these patients wear disposable medical masks or N95 masks (32), avoid taking public transport, and try to stay away from others (at least 1 m). During the medical consultation, patients should fully cooperate with health professionals to identify COVID-19 infection. (3) Health management guidance (33). Good physical function and self-resistance are of great help to prevent COVID-19. Pharmacists should provide patients with health management suggestions, including regular diet, reasonable nutrition, moderate exercise, and adequate sleep and rest during the epidemic. Then, the public can maintain a good nutritional status to reduce the infection risk and improve the disease prognosis. (4) Mental health and psychological support (34). Full attention should be given to the mental health and emotional management of the public at home (especially those suspected to be infected). Pharmacists should ensure that the public properly understands COVID-19 and provide psychological assistance if necessary.

#### Outpatient Visit Period

Outpatient visits are the first line of the health care system in a hospital, and outpatient management is crucial to the entire hospital management system. During the pandemic, in addition to actively participating in the control of in-hospital infections, pharmacists should provide outpatients with more targeted and high-quality patient management, including physician-pharmacist joint clinics, pharmacy clinics, medication therapy management (MTM), medication consultations, drug supply guarantee, and outpatient dispensing services.

##### Control of In-hospital Infections

Since COVID-19 is highly contagious, standard personal protection should be implemented strictly, and prescriptions and other paper documents should be regularly sterilized. Before providing pharmaceutical care to patients, pharmacists must ensure that patients have adopted personal protection and have passed the epidemiological investigation and precheck and triage. During pharmacy services, pharmacists should assist in the identification of suspected patients, timely isolation and referral to fever clinics (6). It is worth mentioning that pharmacists should strengthen awareness of early warnings and indications of self-infection.

##### Physician-Pharmacist Joint Clinic Service

Since regular healthcare services are affected and interfered with, pharmacists need to assist specialists in carrying out care services in a targeted manner. Patient management research of clinical specialists during the COVID-19 pandemic was systematically reviewed and analyzed in Table 1. When providing physician-pharmacist joint clinic services, pharmacists should combine the best recommendations from evidence-based summaries, give full play to their expertise in pharmacy, and assist specialist physicians in providing effective patient management. Taking the hematology clinic for instance, with poor resistance after chemotherapy, patients with hematological malignancy are more susceptible to COVID-19 than the general population and have a worse prognosis with higher mortality after COVID-19 infection (35). In addition, patients with hematological malignancy might have a lower number of white blood cells after conventional infection and have a fever, pneumonia or other symptoms, which are easily confused with COVID-19. However, patients with hematological malignancy and fever have different chest imaging characteristics from those with COVID-19. Therefore, pharmacists should help to educate and comfort patients, ensure that patients with hematological malignancy seek medical attention as soon as possible when they have a fever and other symptoms, make timely differential diagnoses, and provide detailed and practical strategies for personal protection to avoid COVID-19 infection.

##### Pharmacy Clinic Service

When providing pharmacy clinic services, pharmacists should pay attention to patients' epidemiological investigations and symptom monitoring. Since no effective drug has been proved, pharmacists should provide non-pharmaceutical strategies for COVID-19 prevention to patients. Pharmacists should guide patients to understand the epidemic reasonably and take sufficient measures of personal protection while avoiding unreasonable preventive medication or excessive panic. Meanwhile, medication guidance and medication compliance should be strengthened, especially for patients with hypertension, lipid disorders, coronary heart disease, diabetes, asthma and other chronic diseases. These measures can promote safe and rational medication and long-term symptom control and help to reduce the frequency of hospital visits and the risk of COVID-19 infection.

##### Medication Therapy Management

For patients with chronic diseases, the time length of prescription can be properly extended to reduce the frequency of hospital visits. However, as the visit frequency is decreased, improving medication compliance is of greater significance. Furthermore, a rational nutrition diet, regular exercise and healthy rest are also critical to enhance immunity and reduce infection risk. MTM provides not only detailed medication management but also comprehensive lifestyle management (diet, exercise, etc.) as well as continuous follow-up and re-evaluation (36). Pharmacists with MTM qualifications providing integrated and comprehensive MTM services can fully promote rational medication.

##### Medication Consultation Service

Pharmacists should make full use of mobile apps and social media (WeChat and Facebook), phones, texts and other tools to provide online consultations (37). In addition to regular medication guidance, pharmacists should also provide scientific information about COVID-19, including mask wearing, hand hygiene, preparation process of alcohol-containing hand disinfectant, home cleaning and disinfection, and other protective measures. Notably, pharmacists should ensure that patients seek timely medical treatment when COVID-19 infection is suspected and not take drugs on their own following unconfirmed information.

##### Drug Supply Guarantee

During the epidemic, drug supply guarantee is one of the most essential responsibilities of pharmacists (38). For the medical treatment of infected cases, the demand for drugs is urgent and concentrated. Timely, orderly and reasonable drug supply guarantee is of great significance in promoting clinical treatment, promoting epidemic control, and maintaining social stability. Pharmacists should be designated to take charge of the purchase, storage, and distribution of key therapeutic drugs. The appointed pharmacists should adjust the inventory in a timely manner to meet the clinical demand. Key drugs involve antiviral drugs, immunomodulatory drugs, glucocorticoids, antimicrobials, vasoactive drugs, and intestinal micro-ecological regulators, as well as Chinese patent medicine and Chinese herbal medicine (4, 39, 40).

##### Outpatient Dispensing Service

When dispensing drugs, pharmacists should provide personal protection and other COVID-19-related information through oral education and poster displays. Pharmacists should open dispensing windows at intervals to avoid patients gathering and ensure that patients are in line to keep a safe distance (41). Meanwhile, to reduce the frequency of hospital visits, a series of special medical insurance policies have been issued, including extending the time length of prescription and medical insurance of telemedicine. Therefore, pharmacists should strengthen medication instructions and ensure that patients take drugs with doctor advice. Combined with telemedicine, patients can provide home delivery of drugs by express. In addition, pharmacists should encourage patients in various ways to maintain a good mentality and confidence in overcoming the epidemic, such as writing encouraging texts in patient education materials.

#### Hospitalization Period

Confirmed cases need to be admitted to COVID-19-designated hospitals for further inpatient treatment. First, most potential drugs for COVID-19 are still in clinical trials with uncertain safety and efficacy. Second, lopinavir/ritonavir and other antiviral drugs have complicated drug interactions. In addition, some patients may quickly progress to severe or critical condition, with acute respiratory distress syndrome (ARDS), sepsis, and multiple organ dysfunction, particularly in patients with complicated underlying diseases (diabetes, hypertension, cardiovascular disease, malignancy, etc.) (42, 43). Therefore, it is necessary for pharmacists to provide targeted pharmaceutical care for COVID-19 inpatients, including participating in making evidence-based decisions for medication, monitoring and evaluating the safety and efficacy of medications, providing drug interactions management, providing strengthened care for special populations and patients with combined underlying diseases, monitoring and management of convalescent plasma therapy (CPT) and so on (44).

#### Non-hospitalized or Discharged Patients

For COVID-19 patients discharged from hospitals, home isolation and self-monitoring for 14 days are still required. Pharmacists should formulate education materials for discharge medication to ensure medication compliance. Additionally, pharmacists should conduct regular telephone follow-up after discharge. A pharmacy management file for discharged patients should be established, and medication adjustment should be made if necessary. From the Chinese perspective, COVID-19 patients need to go to a designated hospital for follow-up in the 2nd and 4th weeks after discharge (45), so pharmacists should remind patients to complete subsequent visits as planned. Notably, due to positive results of viral nucleic acid retesting in some of the discharged patients, pharmacists should particularly ensure discharged patients to conduct self-monitoring and self-isolation at home. Discharged patients ought to reduce close contact with family members, have meals separately and avoid going out during home staying. Strategies for home-based patient management are in the “Home quarantine period.”

## Discussion

At present, several COVID-19 vaccines (such as Pfizer-BioNTech, Moderna, Johnson and Johnson's Janssen) have been confirmed to be effective and approved, and the U.K. has recently approved molnupiravir (an oral antiviral medication) to fight COVID-19. However, since the genome of the novel coronavirus continues to mutate during the epidemic, the prevention and control of the COVID-19 pandemic is still a challenging difficulty. Thus, medical management and infection control are still issues that must be emphasized. Medical staff play a vital role in epidemic control, while patient management during the epidemic is considerably different from that in the past. Only a multidisciplinary patient management strategy can maintain the proper functioning of the medical system, promote epidemic control, restore the order of life, and reduce the negative impact of the COVID-19 pandemic. Therefore, reasonable and feasible patient management strategies should be developed for doctors, nurses, pharmacists and other medical staff. As pharmacists, we paid more attention to the pharmacist management of the public and patients in the present study. We aim to formulate an integrated entire-process management strategy for pharmacists to join the collaborative force in the COVID-19 pandemic response.

### Recommendations for Pharmacist Management

Based on the evidence-based review of patient management research in major public health emergencies, combined with the practical strategy of Chinese pharmacist management during the COVID-19 period, recommendations were formulated for pharmacists. Regarding the home quarantine period, pharmacist management services shall include medication guidance, guidance on risk monitoring, sanitation measures education, health management guidance and psychological support. Regarding the outpatient visit period, pharmacists should participate in the control of in-hospital infections and provide physician-pharmacist joint clinic services, pharmacy clinic services, medication therapy management, medication consultation services, drug supply guarantee and drug dispensing services. Regarding the hospitalization period, pharmacist management services should include monitoring and evaluating the safety and efficacy of medications, providing strengthened care for special populations and other pharmaceutical care. For non-hospitalized or discharged patients, pharmacist management services should include formulating medication material, conducting telephone follow-up, and establishing pharmacy management files.

### Strengths and Significance

To the best of our knowledge, there have not been any previous studies addressing the management strategy of the public and patients from pharmacists' perspective in the era of COVID-19. Compared with published patient management studies (7–26, 28–30) focusing mostly on doctors and nurses, the current study emphasizes the indispensable role of pharmacists in patient management, exposing and remedying the gaps in the existing patient management. Compared with existing studies on pharmaceutical care, a previous mapping review by our study team (46) demonstrated that the existing studies mostly focused on some specific aspects of pharmaceutical services, such as drug supply, infection control of pharmacists, and online pharmaceutical services. To a certain extent, the existing studies lacked sufficient details to implement patient management for pharmacists in daily work. Overall, the strategy of pharmacist management linked to current clinical management has not yet been established.

Therefore, combining the existing patient management mode, the present study formulated fresh measures of pharmacist management. Pharmacist management measures in key links were strengthened, including prehospital home quarantine, hospitalized medical treatment and follow-up after discharge. Finally, a patient-centered and multidisciplinary-involved strategy of patient management was established and can be put into practice. A previous study illustrated the paradigm shift of drug information centers during the COVID-19 pandemic (47). It emphasized the pharmacists' role in providing information for the public on home care, medication management of patients with chronic comorbid illnesses and psychological support, which were consistent with the present study. Besides, an additional strategy of entire-process management, especially for inpatients and outpatients, was stressed in our study. Similar to our original intention, a previous study partially summarized China's approaches to the control of COVID-19, which pharmacists worldwide can learn from (48).

At present, the COVID-19 outbreak in China has been almost brought under control. However, the global impact of the COVID-19 pandemic is still continuing, and we are still facing the threat and risk of the new waves of global outbreaks. As a member of the health care team, global pharmacists should give full play to their professional advantages and cooperate with physicians and nurses to establish a team-based and optimal patient management mode. Pharmacists should actively carry out pharmaceutical services during the entire process, including the home isolation period, outpatient visit period, hospitalization period and follow-up after discharge. In particular, patient management in weak links should be strengthened, such as self-medication guidance at home, physician-pharmacist joint clinic service, monitoring of medication efficacy and safety, and follow-up of discharged patients. As an increasing number of vaccines hit the market, pharmacists should also strengthen the evaluation of the postmarketing safety and pharmacovigilance of vaccines (49). Then, routine medical work, infection control, COVID-19 patient treatment and other work can be carried out in a scientific and orderly manner.

### Limitations and Future Perspective

Several limitations should be considered for our study. First, the evidence-based review only included published research reports of patient management, while some patient management measures might be published as official documents rather than research papers. Second, the pharmacist management strategy was formulated on the basis of Chinese experience, which has been proved suitable for China's own national conditions. For pharmacists around the world, this strategy should be referred and implemented in light of the actual national conditions of each country. The aforementioned limitations warrant future validation studies into pharmacist management of the public and patients.

## Conclusion

Our study established an evidence-based, patient-centered and entire-process-integrated pharmacist management strategy of the public and patients, in which pharmaceutical care as well as public health services need to be of equal importance. This strategy remedies the gaps in the existing patient management and can be implemented to support pharmacists' contributions to COVID-19 pandemic control.

## Data Availability Statement

The original contributions presented in the study are included in the article/supplementary material, further inquiries can be directed to the corresponding author.

## Author Contributions

ZS and RZ conceived this manuscript and performed the manuscript frame. ZS and YH identified reports of included studies and extracted data. ZS, YH, ZR, GW, and SL performed all statistical analyses, checked for statistical inconsistency, and interpreted the data. ZS and YH drafted the report. SZ, LY, and RZ critically reviewed the manuscript. All authors approved the submitted version.

## Funding

Grant funding from the National Natural Science Foundation of China (72042013) was used to help conduct this research.

## Conflict of Interest

The authors declare that the research was conducted in the absence of any commercial or financial relationships that could be construed as a potential conflict of interest.

## Publisher's Note

All claims expressed in this article are solely those of the authors and do not necessarily represent those of their affiliated organizations, or those of the publisher, the editors and the reviewers. Any product that may be evaluated in this article, or claim that may be made by its manufacturer, is not guaranteed or endorsed by the publisher.
